# Endoscopic Ultrasound and Endoscopic Retrograde Cholangiopancreatography for Bile Duct Stones—Avoiding the Avoidable

**DOI:** 10.3390/biomedicines14010091

**Published:** 2026-01-01

**Authors:** Stefan Chiriac, Catalin Sfarti, Horia Minea, Sebastian Zenovia, Irina Girleanu, Laura Huiban, Cristina Muzica, Adrian Rotaru, Remus Stafie, Robert Nastasa, Ermina Stratina, Bogdan Mihnea Ciuntu, Raluca Avram, Anca Trifan

**Affiliations:** 1 Grigore T. Popa University of Medicine and Pharmacy, 700115 Iasi, Romania; andrei-chiriac@umfiasi.ro (S.C.); cvsfarti@gmail.com (C.S.); sebastianzenovia20@gmail.com (S.Z.); gilda_iri25@yahoo.com (I.G.); lungu.christina@yahoo.com (C.M.); stafieremus@gmail.com (R.S.); robertnastasa948@gmail.com (R.N.); stratina.ermina@yahoo.com (E.S.); bogdan-mihnea.ciuntu@umfiasi.ro (B.M.C.); ralucaioanaavram@gmail.com (R.A.); 2 Institute of Gastroenterology and Hepatology, “St. Spiridon” Emergency Hospital, 700111 Iasi, Romania; ancatrifan@yahoo.com; 3 Department of General Surgery, St. Spiridon” Emergency Hospital, 700111 Iasi, Romania

**Keywords:** endoscopic ultrasound, endoscopic retrograde cholangiopancreatography, choledocholithiasis

## Abstract

**Background:** Endoscopic retrograde cholangiopancreatography (ERCP) is the primary treatment option for choledocholithiasis. However, this procedure carries an inherent non-negligible risk of complications, requiring precise indications and careful patient selection. Endoscopic ultrasonography (EUS) can verify the presence of bile duct stones prior to ERCP. The current ESGE recommendations permit ERCP in high-risk patients without confirmation; however, several individuals undergo ERCP without evident advantage, indicating a necessity for enhanced stratification. **Objectives:** We aim to evaluate the rate of EUS-validated choledocholithiasis in patients with suspected common bile duct (CBD) stones and to determine the predictors of residual stones. A secondary objective was to create and internally validate a streamlined scoring system to enhance risk assessment in ESGE high-risk patients. **Methods:** We conducted a retrospective analysis of patients who had endoscopic ultrasound for suspected choledocholithiasis from January 2023 to December 2024 at a tertiary center. Multivariate logistic regression determined independent predictors of retained calculi. A simplified score was derived from model coefficients and internally validated. **Results:** Among 438 examined patients, 186 were included and 87 had choledocholithiasis confirmed via EUS. ERCP was conducted in 81 patients and postponed for 6 patients due to contraindications. According to the ESGE criteria, 10 patients (5.4%) were classified as low risk, 92 (49.5%) as intermediate risk, and 84 (45.2%) as high risk for choledocholithiasis. For high-risk individuals, EUS identified stones in 45 (53.5%), while 39 (46.4%) experienced spontaneous clearance. Acute pancreatitis (aOR 0.075), cholangitis (aOR 6.939), and EUS CBD diameter (aOR 1.220 per mm) were independent predictors of stones. The resultant three-component score (−2 to +4 points) demonstrated effective discrimination (AUROC 0.788). A criterion of ≥2 resulted in 85.7% sensitivity and 59.0% specificity. **Conclusions:** Almost fifty percent of ESGE high-risk patients were not found to have CBD stones during EUS. Integrating EUS data with a straightforward predictive score may enhance risk classification and avert superfluous ERCP procedures.

## 1. Introduction

Bile duct stones represent one of the most frequently diagnosed conditions. Patients diagnosed with gallbladder stones remain mostly asymptomatic, although 10–20% can develop symptoms and complications [1]. Migration from the gallbladder or primary development of stones into the bile ducts has been associated with cholangitis, acute pancreatitis (AP), and jaundice in up to 10–30% of cases [2]. Moreover, up to 25% of patients diagnosed after cholecystectomy with small bile duct stones that are not removed present a risk of developing complications. Given these observations, most international endoscopy societies recommend ERCP with biliary duct stone removal even in asymptomatic patients, as long as they are fit enough to tolerate this intervention [1,3,4].

Endoscopic retrograde cholangiopancreatography (ERCP) has been the cornerstone of management of common bile duct (CBD) stones since the introduction of this procedure half a century ago [5]. The indication for ERCP shifted from a diagnostic and therapeutic procedure in the years of its inception to only therapeutic after 2000, when pre-therapeutic radiological examinations such as computed tomography (CT) and magnetic resonance cholangiopancreatography (MRCP) became more available. In addition, practitioners became more aware of the need to rigorously select patients that would benefit most from ERCP, in light of data that showed a high risk for complications, mainly post-ERCP pancreatitis (PEP) [6]. Thus, therapeutic ERCP in the absence of confirmation of biliary stones is not currently advisable, as most international endoscopy societies recommend either MRCP or endoscopic ultrasound (EUS) to be performed prior to ERCP if the initial evaluation suggests a risk for CBD stones but abdominal ultrasound (US) or CT do not confirm it. This approach aids in preventing unnecessary ERCPs, hence also reducing unwanted exposure to fluoroscopic radiation [1,3,4].

MRCP has high accuracy for the detection of biliary stones and is currently recommended in patients with a clinical suspicion of choledocholithiasis when other diagnostic modalities are indeterminate [1,3,4]. However, the availability of MRCP is not homogenous among the Member States of the European Union. In countries such as Finland, there are 2.7 MRI units per 100,000 inhabitants, but in countries such as Romania, Latvia, and Slovakia, the rate is much lower, namely 0.4 MRI units per 100,000 inhabitants [7]. These limitations in obtaining a definite diagnostic for choledocholithiasis can lead to unnecessary ERCP procedures being carried out in patients that have had spontaneous CBD clearance. There is significant evidence showing that ERCP should be avoided in patients who no longer have CBD stones, as these patients present the highest risk for PEP, especially in the setting of young age and difficult cannulation [8,9]. Thus, obtaining confirmation of choledocholithiasis before performing ERCP is of paramount importance for decreasing the risk of complications and improving the outcomes.

Endoscopic ultrasound (EUS) was introduced in the late 1970s as a diagnostic technique. It has since evolved, expanding the initial diagnostic yield with pioneering tissue acquisition in digestive and extra digestive tumors [10], as well as therapeutic applications such as biliary stent placement in cases of malignant biliary obstruction not amenable to ERCP, and pseudocyst drainage [11]. Since it was introduced, EUS has become increasingly available, and has been shown to be highly accurate in detecting small CBD stones as well as sludge [12]. Recent data suggest that EUS has an even higher sensitivity than MRI in detecting CBD stones (0.07 for a 95% confidence interval (CI) between 0.91 and 0.99 vs. 0.87 for a 95% CI between 0.80 and 0.93, *p* = 0.006), although comparable specificity (0.90 for a 95% CI between 0.83 and 0.94 vs. 0.92 for a 95% CI between 0.87 and 0.96, *p* = 0.42) [13]. However, EUS presents some disadvantages, as it requires anesthesia and as an endoscopic technique is potentially associated with complications, albeit rarely [14]. Moreover, not all centers in which ERCP is being performed can offer EUS. This is mainly caused by the need for additional training in EUS as well as by the high costs of the equipment used. Thus, the selection of patients that will receive EUS before ERCP has been intensely discussed, and a consensus regarding the optimal course of action has not been reached.

The main objective of this study was to assess the rate of EUS choledocholithiasis confirmation in patients with clinical suspicion of CBD stones and to identify predictors for choledocholithiasis. The secondary objective was to develop and internally validate a simplified clinical prediction score to refine risk stratification in patients with a high risk of choledocholithiasis according to the ESGE criteria.

## 2. Materials and Methods

We performed a retrospective evaluation including procedures performed in the St. Spiridon Emergency Hospital, Institute of Gastroenterology and Hepatology, Iasi, Romania, a tertiary high-volume center.

Consecutive patients that underwent EUS from 1 January 2023 to 31 December 2024 for clinical and/or imagistic suspicion of choledocholithiasis were included (biliary pain, abnormal liver tests, cholangitis criteria, US/CT evidence of CBD stones or dilation). Exclusion criteria were as follows: suspected or confirmed biliary or pancreatic cancer, pregnancy, and incomplete records.

Patients diagnosed with biliary stones were managed according to local expertise as follows: those that fulfilled the ESGE criteria for a low risk of CBD stones (normal liver function tests (LFTs) and no CBD dilation on US) were referred directly to cholecystectomy if gallbladder stones were diagnosed during US. If an intermediate risk of CBD stones was considered (abnormal LFTs and/or CBD dilation on US) then either MRI or EUS was performed, at the discretion of the referring physician. If a high risk of CBD stones was considered (cholangitis, or CBD stones identified via US/CT/MRCP) then ERCP (preceded or not by EUS) was performed at the discretion of the referring physician. EUS was also carried out in cases of clinical suspicion of CBD stones in patients that had previously undergone cholecystectomy. CBD dilation was considered in the case of a diameter > 6 mm in patients that had not undergone cholecystectomy and of >8 mm in patients that had previously undergone cholecystectomy.

Acute cholangitis was defined and classified as grade I, II, and III according to the 2018 Tokyo criteria [15]. AP was diagnosed based on the revised Atlanta criteria [16].

Data concerning demographic information, size of the CBD, presence or absence of CBD stones and/or sludge, time to ERCP, time of hospitalization, outcomes of the patients, and presence of post-EUS and ERCP complications were obtained from the electronic database in a confidential manner. The indication, endoscopic diagnosis, procedures performed, and success rates were also noted. Incomplete records were excluded from the study.

### Statistical Analysis

Data were analyzed using IBM Statistical Package for Social Sciences (SPSS) version 22.0. The distribution of the continuous variables was assessed using the Kolmogorov–Smirnoff test. Variables were expressed as medians (interquartile range, IQR) in the case of variables presenting a nonparametric distribution and means (±standard deviation (SD)) for variables that showed a normal distribution. Categorical variables were expressed as frequencies as well as percentages and statistical significance was analyzed using the chi-squared or Fisher’s test, accordingly. Statistical significance was considered for a *p*-value of less than 0.05.

A subgroup analysis including only high-risk choledocholithiasis patients was performed. The primary outcome was the presence of choledocholithiasis confirmed via EUS. Candidate predictors were selected based on clinical relevance and significance in univariate testing and included AP, cholangitis, CBD diameter on EUS, ultrasound-defined CBD dilation, serum bilirubin, age, and sex. Categorical variables were dichotomized prior to entry. Continuous variables were retained without transformation.

A multivariate logistic regression model using the ENTER method was performed to identify independent predictors of retained CBD stones within the high-risk subgroup. Model calibration was assessed using the Hosmer—Lemeshow goodness-of-fit test, and explanatory power was evaluated using Nagelkerke’s R^2^. Adjusted odds ratios (aORs) with 95% confidence intervals (CIs) were reported, with statistical significance set at *p* < 0.05.

Following the identification of independent predictors, a simplified additive prediction score was derived using the β-coefficients from the final model. Point assignments were scaled according to the magnitude and direction of association. Each patient in the high-risk subgroup was assigned a total score. Internal validation of the score was performed within the same dataset by constructing a receiver operating characteristic (ROC) curve using the score as the test variable and EUS-confirmed choledocholithiasis as the state variable. Discriminative performance was expressed as the area under the ROC curve (AUROC), and sensitivity and specificity estimates were obtained from ROC coordinate tables.

## 3. Results

A total of 438 patients were evaluated for eligibility. After applying the exclusion criteria, 186 patients were included. The general characteristics of the studied group are presented in Table 1. A total of 87 patients (46.8%) were diagnosed with choledocholithiasis and 81 underwent ERCP. In 6 patients ERCP was not performed during the hospital stay because of a lack of emergency and the presence of contraindications, namely coagulation disorders and/or anticoagulant/antiaggregant treatment. In all of these cases, ERCP confirmed the presence of choledocholithiasis and the procedure was successful. There were no EUS-associated complications. However, there were several ERCP-related complications: 3 patients (3.7%) developed PEP and 9 patients (11.1%) presented post-procedural bleeding. There were no procedure-related deaths.

Ninety-nine patients did not have choledocholithiasis, as shown by the EUS examination. When compared to patients with EUS-proven choledocholithiasis, patients with EUS-excluded choledocholithiasis presented a higher percentage of AP 75% vs. 25%, *p* = 0.001, but a lower frequency of cholangitis (43.7% vs. 56.3%, *p* = 0.04). There were significant differences between the groups with EUS-proven and EUS- excluded choledocholithiasis regarding the diameter of the CBD when assessed via US, CT, and EUS: 10 mm (8–12) vs. 7 mm (5.7–10), *p* < 0.001; 12 mm (9.5–13.5) vs. 9.2 (5.2–10.7), *p* = 0.001; and 10 mm (8–13) vs. 6 mm (5–8), *p* < 0.001, respectively (Table 2).

AP was significantly less common among patients with CBD stones (12.6% vs. 33.3%) and was inversely associated with choledocholithiasis (χ^2^ = 10.98, *p* = 0.001; OR = 0.29, 95% CI: 0.14–0.62). These findings suggest lower odds of retained stones in these cases. Cholangitis was more frequent among patients with confirmed CBD stones (χ^2^ = 4.22, *p* = 0.040; OR = 1.87, 95% CI: 1.03–3.40). Dilated CBD on ultrasound was strongly associated with EUS-confirmed stones (85.1% vs. 55.6%; χ^2^ = 18.96, *p* < 0.001; OR = 2.52, 95% CI: 1.53–4.15).

In the subgroup of patients where CT was performed (n = 67), CBD dilation was associated with the presence of CBD stones (95.5% vs. 73.3%; χ^2^ = 4.62, *p* = 0.032; Fisher’s *p* = 0.047), though the odds ratio did not reach significance due to a wide confidence interval (OR = 5.06, 95% CI: 0.75–34.23).

### 3.1. Subgroup Analysis of Patients with AP

Although without statistical significance, we found a higher proportion of women in the choledocholithiasis group compared to the group without CBD stones (81.8% vs. 51.5%, *p* = 0.077). 

**Figure 1:**
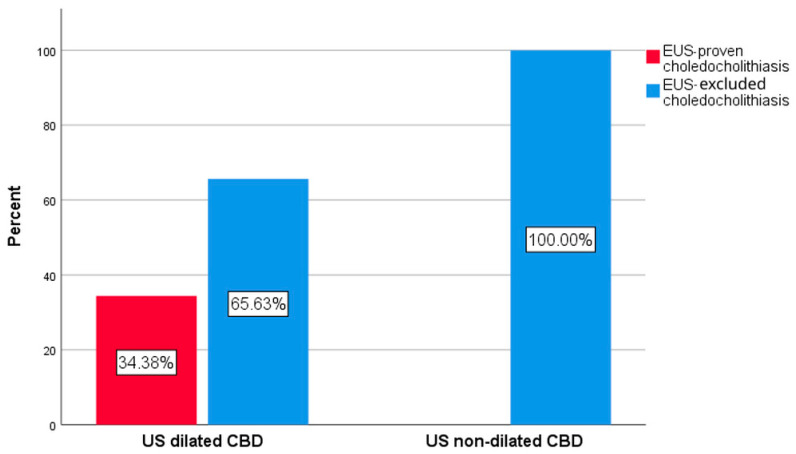
The rate of EUS-proven choledocholithiasis in patients with AP according to US biliary dilation of the CBD.

Concerning the 44 patients that presented AP, 25 (56.8%) also presented acute cholangitis according to the Tokyo 2018 criteria. In this subgroup, EUS examination showed the presence of CBD stones in 7 (28%) of the cases. Among the patients with AP, 12 (27.3%) did not present dilated CBD during the initial US examination. None of the patients presenting AP and non-dilated CBD during US had confirmed CBD stones during EUS, while in the case of patients with CBD US dilation, 21 (65.6%) presented spontaneous CBD clearance as confirmed by negative EUS (Table 3, Figure 1).

Univariate analysis indicated an OR of not having CBD stones if CBD was dilated of 0.656, 95% CI (0.511–0.843), *p* = 0.02.

### 3.2. Subgroup Analysis of Patients with a High Risk for Choledocholithiasis

A subgroup of 84 patients presented a high risk for CBD stones according to the ESGE criteria. However, 39 (46.4%) of these presented no stones when EUS examination was performed.

There were 84 patients that fulfilled the high risk of choledocholithiasis ESGE criteria (Table 4). Among these, 45 (53.5%) had EUS-proven choledocholithiasis and 46.4% did not have choledocholithiasis according to the EUS examination. There were no statistically significant differences concerning sex or age between the two groups. However, from this subgroup of 84 patients, 56% of the patients with no proven EUS lithiasis were diagnosed with AP compared to only 15.6% of those with choledocholithiasis (*p* < 0.001).

Univariate analysis identified AP and cholangitis as risk factors for the presence of choledocholithiasis as assessed via EUS in patients fulfilling high-risk criteria for choledocholithiasis: OR = 2.862, 95% CI (1.466–5.588), *p* = 0.002; OR = 1.670, 95% CI (1.080–2.581), *p* = 0.047, respectively. In cases without dilatation of the CBD, there was a higher probability of not having choledocholithiasis: OR = 1.670, 95% CI (1.080–2.581), *p* = 0.047.

Multivariate logistic regression identified three independent predictors of retained CBD stones in this high-risk subgroup of patients. AP was inversely associated with choledocholithiasis (B = −2.595, *p* < 0.001; aOR 0.075, 95% CI 0.019–0.297). Cholangitis was independently associated with an increased likelihood of CBD stones (B = 1.937, *p* = 0.015; aOR 6.939, 95% CI 1.454–33.116). CBD diameter measured during EUS was also a significant continuous predictor (B = 0.199, *p* = 0.027; aOR 1.220, 95% CI 1.023–1.454). Ultrasound-defined CBD dilation, bilirubin level, age, and sex were not significant in the adjusted model.

The logistic regression demonstrated good calibration (Hosmer–Lemeshow χ^2^ = 4.358, df = 8, *p* = 0.823) and satisfactory explanatory performance (Nagelkerke R^2^ = 0.448).

#### Scoring System

**Figure 2:**
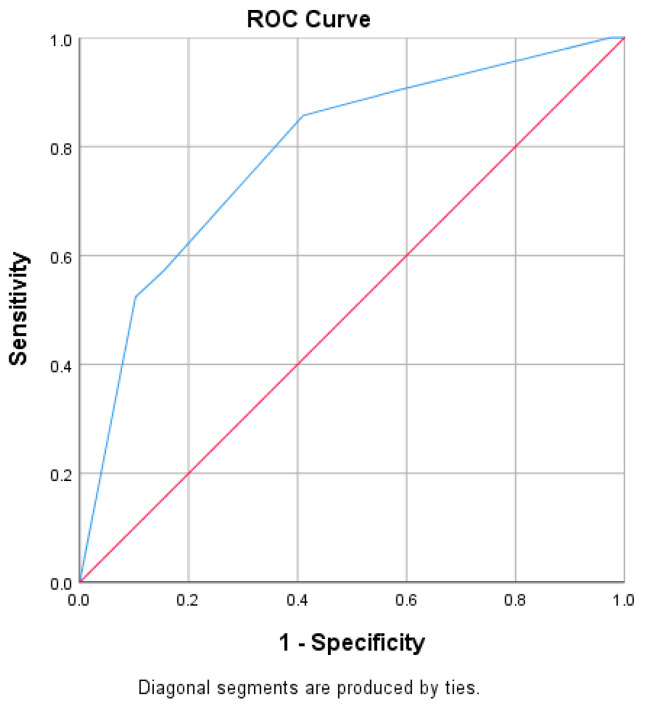
ROC analysis of the EUS-CBD predictive score for choledocholithiasis.

We developed a scoring system based on these predictors. This score comprised 3 elements ranging from −2 to +4 points (−2 for AP, +2 for cholangitis, +1 for CBD diameter 8.0–9.9 mm, and +2 for CBD diameter ≥10 mm) (Table 5). Internal validation showed good discriminative ability, with an AUROC of 0.788 (Figure 2). A threshold of ≥2 points provided the optimal balance between sensitivity and specificity (85.7% and 59.0%, respectively). Higher thresholds increased specificity (e.g., ≥3 points: specificity 89.7%) at the expense of reduced sensitivity (52.4%).

## 4. Discussion

International guidelines suggest that patients presenting with high-risk criteria for choledocholithiasis should undergo ERCP even in the absence of definite pre-ERCP imagistic confirmation [1,3,4,17]. Patients with suspected choledocholithiasis are stratified into low, intermediate, and high risk, according to ESGE recommendations. Even though these recommendations are applicable to a vast proportion of patients, there are still some, even with high-risk criteria prompting for ERCP, that will present spontaneous CBD clearance and thus be exposed to ERCP-related complications without potential benefit. In our analysis we showed that from the subgroup of 84 patients that fulfilled high-risk criteria for choledocholithiasis, in 39 (46.4%) of them the presence of CBD stones was not confirmed during the EUS examination, and thus those patients did not undergo ERCP.

**Figure 3:**
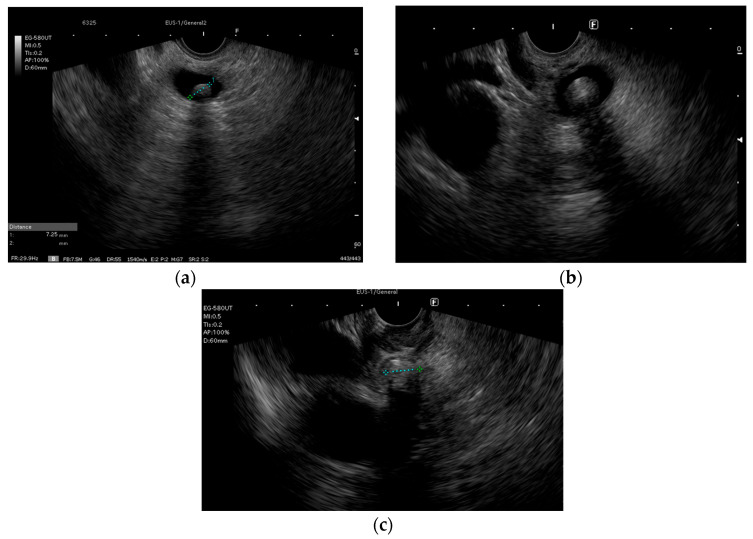
(**a**) Endoscopic ultrasound diagnostic of choledocholithiasis; (**b**) infracentimetric stones in the common bile duct; (**c**) stone impacted near the papilla in the common bile duct.

Regarding the best method of examination, EUS has recently been shown to present higher accuracy for the diagnosis of CBD stones, as demonstrated by Varone et al. [18] in a retrospective study comprising 736 patients from the Eastern Association for the Surgery of Trauma (EAST) Retained Common Bile Duct Stone database. The authors found that in patients that fulfilled the ASGE intermediate risk criteria for CBD stones, EUS had a positive predictive value (PPV) of 90.5% and a negative predictive value (NPV) of 95.6%, with a 95% CI (76–99.8%), while MRCP had a PPV of 74.4% and an NPV of 79.2%, with a 95% CI (71–85.6%). Our results show that in patients with a moderate risk of choledocholithiasis, EUS identified CBD stones in 41 cases (44.6%), while in the high-risk group 45 patients (53.5%) had EUS-confirmed choledocholithiasis (Figure 3). These findings confirm the usefulness of performing EUS in intermediate-risk patients and are in accordance with general good practice recommendations. Both the ESGE and the 2019 ASGE recommendations approve the use of EUS in patients with an intermediate risk of choledocholithiasis [1,17]. In high-risk patients, as per the 2019 ASGE criteria, there is a lack of homogenous data regarding the rate of confirmed CBD stones. Real-life studies assessing the accuracy of these criteria show a variable percentage of patients with a high risk for CBD stones who do not have confirmed lithiasis (17–57%) [18,19,20,21]. Thus, there are advocates for performing EUS, where available, regardless of the allocation of the patients to high-risk criteria groups, unless they present an unequivocal stone on imaging [20,22]. Our results support this view, as almost half of our high-risk patients did not have CBD stones and thus would have received ERCP and consequently been exposed to procedure-associated risks without a benefit.

AP presents a high risk for unfavorable outcomes, warranting that urgent ERCP should be performed, especially in the setting of cholangitis, when most societies recommended undertaking biliary drainage in the first 12–72 h [1,3,4,15,17,23]. However, recent guidelines no longer include isolated AP as a criterion for the definition of patients with a high risk of choledocholithiasis, owing to conflicting data [4]. Patients who present AP as well as cholangitis should, however, be considered for urgent ERCP. Currently, the definition of cholangitis and the grading of severity follow the Tokyo 2018 criteria. These have been validated to confirm the diagnosis, as well as to grade the severity of cholangitis and thus to aid in the implementation of timely biliary drainage [15]. The 2018 Tokyo criteria include systemic inflammation, manifested by elevated or very low white blood cells (WBCs) and/or elevated CRP, high bilirubin levels, abnormal liver enzymes, and imagistic evidence of biliary dilatation or etiology. However, in the case of AP associated with spontaneous CBD clearance, elevated WBC and CRP can be found in the absence of cholangitis, and elevated bilirubin levels together with abnormal liver enzymes are frequently associated [24]. Thus, these patients can be considered at high risk of choledocholithiasis as per the current guideline recommendations and therefore receive ERCP without a definitive confirmation of CBD stones. These diagnostic particularities could explain the low rate of CBD stones encountered in our patients from the high-risk group. We found that 18 (72%) of the patients presenting with AP and cholangitis according to the 2018 Tokyo criteria had spontaneous CBD clearance, as EUS examination did not show CBD stones. Thus, ERCP was avoided in these patients. These findings are of clinical importance, as efforts should be made to limit the use of ERCP in cases where there is no clear benefit for the patient [8].

Current ESGE recommendations suggest that in patients with suspected biliary stricture and/or unexplained CBD dilatation, EUS should be performed, even in the absence of abnormal laboratory tests. The rationale is based on the high accuracy of EUS for the detection of choledocholithiasis, combined with the rare complications associated with this diagnostic procedure, comparable with those of diagnostic upper gastrointestinal endoscopy [25]. However, in patients classified as having a high risk of choledocholithiasis, recent guidelines suggest proceeding directly to ERCP without pre-procedural confirmation. Nevertheless, nearly half of the high-risk patients in our cohort did not have CBD stones confirmed via EUS, indicating that guideline-defined high risk lacks sufficient specificity in real-world practice and may expose patients to avoidable ERCP-related complications. These findings are in accordance with several studies that show variable stone confirmation rates within the high-risk category. Silva-Santisteban et al. [19] performed a prospective study including 359 patients and compared the accuracy of 2010 and 2019 ASGE criteria and ESGE criteria for the risk of choledocholithiasis. The authors found that in high-risk patients there were variable rates of confirmed choledocholithiasis (83.1% for ESGE criteria, 79.1% for 2010 ASGE criteria, and 81.6% for 2019 ASGE criteria). Similarly, in a retrospective analysis comprising 267 patients with suspected choledocholithiasis, Jacob et al. identified 79% of the high-risk patients according to the 2019 ASGE criteria that had confirmed CBD stones [21].

In our study, within the high-risk for choledocholithiasis subgroup, we identified AP as a strong inverse predictor of retained stones, supporting evidence that spontaneous CBD clearance is common in biliary pancreatitis, particularly in the absence of ongoing obstruction. Conversely, cholangitis was independently associated with the presence of CBD stones.

We proposed a predictive score for the presence of choledocholithiasis in high-risk patients, which was internally validated and demonstrated good performance, with an AUROC of 0.788. We suggest that this score should be used to assist in refining decision-making within the high-risk population. A threshold of ≥2 points identifies patients at significantly higher likelihood of retained stones, whereas lower scores correspond to a low probability and may justify postponing or avoiding ERCP when EUS or MRCP is available. Nonetheless, as the score was developed and evaluated within the same retrospective cohort, overfitting remains possible. External and preferably prospective validation is required before clinical implementation, and the score should presently be regarded as hypothesis-generating.

Our study has several limitations. The study was retrospective and contained a relatively limited sample size, which could lead to risks of selection and information bias. The single-center analysis could also limit the external generalization of the results. Further, the high proportion of patients from the ESGE high-risk subgroup that did not have confirmed CBD stones could be related to local conditions, such as imaging quality or temporal factors influencing stone migration, and thus may not be entirely representative for other populations. Formal calibration plots for the generated score were not created, and external validation is necessary to further evaluate calibration and generalizability. The predictive score was internally validated, and external prospective validation should be performed before implementation in clinical practice.

Overall, these findings support a selective rather than automatic use of ERCP in ESGE high-risk patients and suggest that the integration of EUS-based parameters may improve procedural appropriateness while reducing risk exposure. We suggest that in the absence of biliary dilation, patients with PA and grade 1 cholangitis should undergo EUS rather than ERCP, as spontaneous CBD clearance is common in these patients.

## 5. Conclusions

Even though the high-risk criteria for CBD stones allow for an adequate assessment of patients that would benefit from ERCP, in selected cases such as patients with AP and/or cholangitis without CBD dilation, a pre-procedural EUS examination may prove spontaneous CBD clearance, and thus, an unnecessary ERCP could be avoided in these patients. Our simple scoring system could be used to further stratify the risk of CBD stones and consequently the need for EUS in high-risk patients. However, more randomized large-population multicentric studies may be needed in order to confirm these findings and to externally validate our proposed score. Both MRCP and EUS can be used to confirm the presence of CBD stones before performing ERCP, but EUS has the advantage of immediacy and the option to proceed to ERCP in the same session. Thus, in light of these results, we recommend that in all of the cases where a stone is not seen on cross-sectional imaging studies, an EUS be performed, preferably in the same session as ERCP.

## Figures and Tables

**Table 1:** General characteristics of the studied group.

Characteristic	Value
Age, median (IQR)	60 (43–73)
Sex, women/men, n (%)	117 (62.9)/69 (37.1)
Acute pancreatitis, n (%)	44 (23.7)
Cholangitis, n (%)	71 (38.2)
Grade I	51 (27.4)
Grade II	15 (8.1)
Grade III	5 (2.7)
US CBD diameter, median (IQR)	9 (1–11)
CT CBD diameter, median (IQR)	10 (9–12)
EUS CBD diameter, median (IQR)	8 (6–11)
ERCP CBD diameter, median (IQR)	12 (9–14)
US choledocholithiasis, n (%)	1 (0.5)
CT choledocholithiasis, n (%)	12 (6.5)
EUS choledocholithiasis, n (%)	87 (46.8)
Lithiasis risk, n (%)	
Low risk	10 (5.4)
Intermediate risk	92 (49.5)
High risk	84 (45.2)
ERCP, n (%)	81 (43)
Same-session EUS and ERCP, n (%)	66 (81.5)
Separate-session EUS and ERCP, n (%)	15 (18.5)
ERCP-related complications, n (%)	
PEP	3 (3.7)
Bleeding	9 (11.1)
ALT U/L, median (IQR)	126 (54–318)
AST U/L, median (IQR)	81 (36–211)
ALP U/L, median (IQR)	177 (109–325)
GGT U/L, median (IQR)	331 (120–520)
Bilirubin mg/dL, median (IQR)	2 (0.87–5.11)
Lipase U/L, median (IQR)	45 (26–123)

**Table 2:** Characteristics of patients with EUS-proven and EUS-excluded choledocholithiasis.

Characteristic	EUS-Proven Choledocholithiasis, n = 87	EUS-Excluded Choledocholithiasis n = 99	*p*-Value
Age, median (IQR)	66 (43–74)	59 (42–72)	0.468
Sex, women/men, n (%)	61 (52.1)/26 (37.7)	56 (47.9)/43 (62.3)	0.056
Acute pancreatitis, n (%)	11 (25)	33 (75)	0.001
Cholangitis, n (%)	40 (45.9)	31 (31.3)	0.04
Grade I	29 (72.5)	22 (70.9)	
Grade II	8 (20)	7 (22.5)	
Grade III	3 (7.5)	2 (6.4)	
US CBD diameter, median (IQR)	10 (8–12)	7 (5.7–10)	<0.001
US dilated CBD, n (%)			
CT CBD diameter, median (IQR)	12 (9.5–13.5)	9.2 (5.2–10.7)	0.001
EUS CBD diameter, median (IQR)	10 (8–13)	6 (5–8)	<0.001
US choledocholithiasis, n (%)	1 (100)	0	0.451
CT choledocholithiasis, n (%)	7 (58.3)	5 (41.7)	0.098
Lithiasis risk, n (%)			
Low risk	1 (10)	9 (9)	
Intermediate risk	41 (44.6)	51 (55.4)	
High risk	45 (53.6)	39 (46.4)	
ALT, U/L, median (IQR)	114.5 (43.5–330)	129 (58–273)	0.497
AST U/L, median (IQR)	87 (29.7–212.2)	73 (40–208)	0.203
ALP U/L, median (IQR)	201 (107.2–350.7)	167 (110–290)	0.600
GGT U/L, median (IQR)	278.5 (88.7–575.2)	346 (120–496)	0.327
Bilirubin mg/dL, median (IQR)	2.24 (0.8–5.3)	2 (0.9–4.7)	0.600
Lipase U/L, median (IQR)	30.5 (20.7–60.5)	68 (36–841)	<0.001
WBC × 10^9^/L, median (IQR)	8.6 (7.3–11.8)	10.4 (8–14)	0.042
CRP mg/dL, median (IQR)	1.7 (0.7–2.2)	2 (0.65–9.7)	0.042
Hospitalization days, median (IQR)	8 (5–11)	8 (5–10)	0.468
Creatinine mg, median (IQR)	0.7 (0.6–0.9)	0.78 (0.6–1)	0.197

**Table 3:** Subgroup analysis of patients diagnosed with acute pancreatitis with EUS-proven and EUS-excluded choledocholithiasis.

Characteristic	EUS-Proven Choledocholithiasis, n = 11	EUS-Excluded Choledocholithiasis n = 33	*p*-Value
Age, median (IQR)	71 (24–77)	58 (52–70)	0.99
Sex, women/men, n (%)	9 (81.8)/2 (18.2)	17 (51.5)/16 (48.5)	0.077
Cholangitis, n (%)	7 (63.6)	18 (54.5)	
Grade I	3 (42.8)	13 (72.2)	
Grade II	4 (57.1)	4 (22.2)	
Grade III	0	1 (5.5)	
US CBD diameter, median (IQR)	7 (7–14)	5.2 (4–8.2)	0.803
US non-dilated CBD, n (%)	0 (0)	12 (36.4)	0.02
CT CBD diameter, mean ± SD	12.5 ± 3.5	10.2 ± 3.8	0.422
EUS CBD diameter, median (IQR)	7.5 (7–12)	4.2 (2.6–6)	0.014
US choledocholithiasis, n (%)	0	0	NA
CT choledocholithiasis, n (%)	1 (9)	1 (3)	0.105
Lithiasis risk, n (%)			0.854
Low risk	0	0	
Intermediate risk	4 (36.4)	11 (33.3)	
High risk	7 (63.6)	22 (66.7)	
ALT, U/L, mean ± SD	167.6 ± 104.5	249.6 ± 191.9	0.084
AST U/L, mean ± SD	147.4 ± 167.2	213 ± 192.1	0.319
ALP U/L, mean ± SD	226.7 ± 200	286 ± 183	0.368
GGT U/L, mean ± SD	326 ± 236.8	468.9 ± 345.3	0.211
Bilirubin mg/dL, mean ± SD	4.3 ± 2.8	3.4 ± 2.8	0.340
Lipase U/L, median (IQR)	106 (18–302)	571.7 (217.4–1956.5)	0.037
WBC × 10^9^/L, median (IQR)	4.3 (3.9–5.5)	8.5 (6.9–11)	0.005
CRP mg/dL, median (IQR)	0.26 (0.13–0.86)	0.89 (0.43–3.6)	0.164
Hospitalization days, median (IQR)	8 (6.5–10.5)	8 (6.2–8)	0.340
Creatinine mg, mean ± SD	0.8 ± 0.3	3.8 ± 1.7	0.573

NA, not applicable.

**Table 4:** Subgroup analysis of patients presenting high-risk criteria for choledocholithiasis with EUS-proven and EUS-excluded choledocholithiasis.

Characteristic	EUS-Proven Choledocholithiasis, n = 45	EUS-Excluded Choledocholithiasis n = 39	*p*-Value
Age, median (IQR)	67 (43–75)	54 (37–67)	0.178
Sex, women/men, n (%)	30 (66.7)/15 (33.3)	18 (46.2)/21 (53.8)	0.058
Pancreatitis, n (%)	7 (15.6)	22 (56.4)	<0.001
Cholangitis, n (%)	40 (88.8)	28 (71.7)	0.047
Grade I	29 (72.5)	21 (75)	
Grade II	8 (20)	5 (17.8)	
Grade III	3 (7.5)	2 (7.1)	
US CBD diameter, median (IQR)	10 (7–12)	8.5 (5.8–11)	0.108
US non-dilated CBD, n (%)	5 (11.1)	12 (30.8)	0.056
CT CBD diameter, median (IQR)	12 (11–13)	9.5 (7–12)	0.002
EUS CBD diameter, median (IQR)	10 (9–13)	6.4 (5–8)	0.001
US choledocholithiasis, n (%)	0	0	NA
CT choledocholithiasis, n (%)	5 (31.3)	3 (17.6)	0.438
ALT U/L, median (IQR)	234 (78–330)	272 (147–365)	0.99
AST U/L, median (IQR)	120 (84–294)	214 (68–322)	0.382
ALP U/L, median (IQR)	376 (257–583)	254 (166–364)	0.99
GGT U/L, median (IQR)	430 (254–1322)	413 (347–620)	0.189
Bilirubin mg/dL, median (IQR)	6.2 (2.7–9.8)	4.5 (2.3–6.7)	0.662
Lipase U/L, median (IQR)	25 (15–31)	279 (31–2080)	<0.001
WBC × 10^9^/L, median (IQR)	8.5 (6.6–10)	11.6 (7.1–14)	0.382
CRP mg/dL, median (IQR)	1.9 (1–3.1)	3.1 (0.8–9.1)	0.382
Hospitalization days, median (IQR)	8 (6–9.5)	8 (6–10)	0.817
Creatinine mg, median (IQR)	0.7 (0.5–1.1)	0.7 (0.6–1)	0.662

NA, not applicable.

**Table 5:** Proposed EUS-CBD predictive score for choledocholithiasis in the high-risk group.

Variable	Definition	Assigned Points
Acute pancreatitis	Present at admission or during evaluation	−2
Cholangitis	Clinical cholangitis (Tokyo 2018 criteria), any grade	+2
CBD diameter on EUS	<8 mm	0
	8.0–9.9 mm	+1
	≥10 mm	+2
Suggested risk interpretation		
Total Score	Estimated Probability of CBD Stones	Clinical Implication
≤0 (Low risk)	Very low likelihood	Avoid ERCP; EUS or observation appropriate
1–2 (Intermediate risk)	Moderate likelihood	EUS recommended; ERCP only if confirmed
≥3 (High risk)	High likelihood	Consider same-session EUS → ERCP

## Data Availability

The data used in this study were obtained from the St. Spiridon Emergency Hospital, Institute of Gastroenterology and Hepatology, Iasi, Romania. The database is available upon request from the corresponding authors.

## Abbreviations

The following abbreviations are used in this manuscript:

CBD	Common bile duct
ERCP	Endoscopic retrograde cholangiopancreatography
EUS	Endoscopic ultrasound
ESGE	European Society for Gastrointestinal Endoscopy
ASGE	American Society for Gastrointestinal Endoscopy
PPV	Positive predictive value
NPV	Negative predictive value
OR	Odds ratio
CI	Confidence interval
ROC	Receiver operating characteristic
IQR	Interquartile range
AP	Acute pancreatitis
CT	Computed tomography
US	Ultrasonography
MRCP	Magnetic resonance cholangiopancreatography
AST	Aspartate aminotransferase
ALT	Alanine aminotransferase
GGT	Gamma-glutamyl transferase
ALP	Alkaline phosphatase
WBC	White blood cells
